# Stigma and Ebola survivorship in Liberia: Results from a longitudinal cohort study

**DOI:** 10.1371/journal.pone.0206595

**Published:** 2018-11-28

**Authors:** Luc Overholt, David Alain Wohl, William A. Fischer, Daniel Westreich, Sam Tozay, Edwina Reeves, Korto Pewu, David Adjasso, David Hoover, Carson Merenbloom, Harrietta Johnson, Gerald Williams, Tonia Conneh, Joseph Diggs, Alexandria Buller, Darrius McMillian, Darrel Hawks, Karine Dube, Jerry Brown

**Affiliations:** 1 School of Medicine, The University of Michigan Medical School, Ann Arbor, Michigan, United States of America; 2 The Institute of Global Health and Infectious Diseases, The University of North Carolina, Chapel Hill, North Carolina, United States of America; 3 Eternal Love Winning Africa Hospital, Paynesville, Liberia; 4 Clinical Research Management, Inc., Hinckley, Ohio, United States of America; 5 College of Arts and Sciences, Abilene Christian University, Abilene, Texas, United States of America; Johns Hopkins School of Public Health, UNITED STATES

## Abstract

**Background:**

Survivors of the 2014–2016 West Africa Ebola epidemic have been reported to suffer high levels of stigmatization after return to their communities. We sought to characterize the stigma encountered by a cohort of Ebola survivors in Liberia over time.

**Methods:**

Ebola-related stigma was assessed from June 2015 to August 2017 in 299 adolescent and adult Liberian Ebola Survivor Cohort participants at three month intervals using adapted HIV stigma scales scored from 0 to 10 according to the proportion of answers indicating stigmatization.

**Findings:**

The median time from Ebola Virus Disease (EVD) to study entry was 393 days (IQR 336–492). Participants (43% female) had a median age of 31 (IQR 25–40) years. Mean self-reported stigma levels were greater at baseline (6.28 ± 0.15 [IQR: 4.38–8.75]) compared to the first post-baseline visit (0.60 ± 0.10 [IQR: 0–0]; p<0.0001). During follow-up, stigma levels were stable. Baseline stigma significantly increased during enrollment and following clusters of Ebola re-emergence in Liberia. Survivors encountered primarily enacted and perceived external stigma rather than internalized stigma.

**Conclusions:**

Ebola-related stigma was prevalent among Liberian survivors more than a year after EVD recovery. Self-reported stigma was greater in the period before cohort enrollment; however, some degree of stigmatization persisted years after EVD. Transient rises in stigma were observed following episodic Ebola re-emergence of EVD in Liberia. During future EVD outbreaks, enhanced public health interventions designed to prevent and mitigate Ebola-related stigma that is enacted and external should be implemented to support survivor recovery and community re-integration.

## Introduction

The unprecedented 2014–2016 Ebola epidemic in West Africa killed more than 11,000 people but also left more than 17,000 survivors, [1] many afflicted by both lingering physical symptoms and the mental health consequences associated with surviving Ebola Virus Disease (EVD).[2] Ebola’s effects on mental health can be profound and are thought to stem from the trauma of experiencing EVD and the Ebola Treatment Unit (ETU) environment, grief over lost family members, survivor guilt, and stigmatization.[2–5]

A stigma is an attribute that discredits an individual and precludes their full acceptance in society.[6] The stigma attached to Ebola survivors, largely born of fear of contagion, has led to evictions, intimate partnership dissolution, termination of employment, abandonment, and physical violence.[7–10] These prejudicial and discriminatory manifestations of a stigma are termed enacted stigma.[11] Stigmatized groups also endure perceived external stigma, the perception that they are devalued by society, and the anticipation of enacted stigma in the future.[12] Internalized stigma occurs when the stigmatized themselves endorse the negative views attributed to their stigma.[11] Surveys of Ebola survivors conducted in Guinea during the outbreak suggest that at least some level of stigmatization was nearly ubiquitous.[13] Mixed results have been reported from Sierra Leone, with one survey reporting pervasive stigmatization of survivors,[14] while another found that only 32% of participants reported stigmatization.[5] In these studies, the stigma reported by survivors was generally manifest by the reactions of others to them (e.g., social distancing, verbal abuse). In Liberia, focus groups of Monrovian survivors also reported severe enacted and external stigmatization.[9] These studies included participants who were, generally, within months of ETU discharge and most have focused on enacted stigma without evaluating other types of stigma.

The objectives of the present study were twofold. First, we aimed to characterize stigma attached to Ebola survivors and how perceived stigmatization changed overtime. Secondarily, we sought to categorize the type of stigma faced by Liberian survivors of the West African Ebola epidemic.

## Methods

### Participants and setting

The on-going Longitudinal Liberian Ebola Survivor Study is based at the Eternal Love Winning Africa (ELWA) Hospital in Monrovia, Liberia, the location of two large ETUs during the outbreak. Recruitment began in June 2015 and was completed in June 2016. Those with a history of prior EVD as evidenced by a discharge certificate from an ETU verified by photo identification, at least 5 years of age, and willing and able to consent to participation (for minors, parental assent was obtained) were recruited from the ELWA Ebola Survivor Clinic and the local communities via word of mouth. Stigma was only assessed in participants who were 14 years of age and older as there are limited data regarding the assessment of disease-related stigma among younger children, especially in West Africa, and the adapted HIV stigma scales were developed for adults. In addition, the number of children enrolled in this cohort was small.

Study visits were scheduled every three months. At the visits, study staff administered a questionnaire that covered physical and mental health status and a 16-question survey of Ebola-related stigma. Survey questions explored enacted, internalized, and perceived external stigma, as well as disclosure fears (Table 1). The three possible responses to survey items were disagree, agree, and no response. At baseline, we asked participants about stigma experienced since ETU discharge; during follow-up visits participants were asked to only consider the period that had occurred since the last study visit. All surveys were conducted in private rooms in Liberian English by research assistants who had received training in Good Clinical Practice (GCP) and at least four hours of survey administration, and who successfully completed Collaborative Institutional Training Initiative (CITI) ethics and compliance certification. Participants received $US30 for completion of the study visit, which included completing surveys, physical examination, and blood collection.

**Table 1:** Stigma survey items and source.

Survey question		Source (adapted from)
Enacted stigma		
1	I have been hurt by how people reacted to learning I had Ebola.	Wright et.al
2	I have stopped socializing with some people because of their reactions of my having had Ebola.	Wright et. al
3	I have lost friends because I had Ebola.	Wright et. al
Disclosure concerns		
4	I am very careful who I tell that I had Ebola.	Wright et. al
5	I worry that people who know I have had Ebola will tell others.	Wright et. al
Internalized stigma		
6	I fell that I am not as good as a person as others because I had Ebola.	Wright et. al
7	Having had Ebola makes me feel unclean.	Wright et. al
8	Having Ebola makes me feel that I am a bad person	Wright et. al
Perceived external stigma		
9	Most people think that a person who has had Ebola is disgusting.	Wright et. al
10	Most people are afraid of a person who has had Ebola.	The Authors
11	Most people who have had Ebola are rejected when others find out.	Wright et. al
12	People I know would treat someone who has had Ebola as an outcast.	Wolitski et. al
13	People I know would be uncomfortable around someone who has had Ebola	Wolitski et. al
14	People I know would believe that a person who has had Ebola is dirty	Wolitski et. al
15	People I know would reject someone who has had Ebola	Wolitski et. al
16	People I know would not want someone who has had Ebola around their children.	Wolitski et. al

Note: Stigma category headings were not included in the administered survey and the items were not grouped by stigma category.

The Ebola-related stigma questionnaire was derived from Berger’s HIV stigma scale, a validated measure of self-reported stigma in individuals infected with HIV in many cultural settings.[12, 15–18] A revision of Berger’s scale by Wright et al sought to reduce participant burden by shortening the measure from 40 to 10 items. When developing the scale, the 10 items from Wright’s abbreviated scale that loaded most strongly into each of the four factors (enacted, internalized, and perceived external stigma and disclosure concerns) in Berger’s original factor analysis were included.[16] As has been done previously, we adapted the scale for use in a disease state other than HIV.[19] In addition, the scale was supplemented with items developed by Woltiski et al (also derived from Berger’s scale) that were felt to probe for Ebola-related stigmatization reported from Liberia.[12] To ensure survey items were appropriate and understandable in the Liberian setting, the Liberian research staff and partners participated in the development of the final stigma survey, reviewed the survey and made simple wording edits following mock encounters in which the survey was administered to other Liberian research staff members.

All participants provided written informed consent with assent obtained from minors age 14 to 18 years and consent from their parent/guardian. The consent document and the research protocols were approved by the Institutional Review Boards at the University of North Carolina at Chapel Hill and the University of Liberia—Pacific Institute of Research (UL-PIRE).

### Analytic considerations

#### Scoring and characterizing stigma

A total stigma score was derived for each participant at each visit and was calculated as the proportion of all answered questions that indicated stigmatization multiplied by ten. Unanswered questions were not included in the denominator (there were few instances of unanswered questions). Scores for the subscales were likewise calculated as the proportion of the subscale responses that indicated stigmatization multiplied by ten (and so individuals received scores between 0 and 10 corresponding to the proportion of yes answers out of all questions for which a yes or no answer was given; answers of “no response” were not counted in estimating this score). A one-way ANOVA was used to compare responses indicating stigmatization (the one-way factor) between four subscales (enacted stigma, disclosure concerns, internalized stigma and perceived external stigma). When differences were detected, means were separated using Tukey’s test. These are standard statistical methods for comparing means between multiple datasets when there is only one factor involved. Tukey’s was selected among other means comparison procedures because it is typically used in medical research and it maintains the type I error probability (finding a difference where none exists) at the selected probability level (P<0.05 in our case). The analysis was repeated to both classify the type of stigma faced at baseline visits, and at follow-up visits. All measures of central tendency are reported as means ± standard error of the mean.

#### Stigma at baseline vs. follow-up visits

During the baseline visit, participants considered all events that had occurred since ETU discharge. Baseline stigma scores were compared to the scores at the next follow-up visit. Means were compared using two sample t-test assuming unequal variance. These, in turn, were compared to the scores obtained at the remaining subsequent study visits using an ANOVA followed by post-hoc Tukey’s test.

#### Temporal variations in stigma at baseline

The June 2015 to June 2016 period of enrollment was divided into four equal time spans (quartiles), each of 78 days; we compared mean baseline stigma score for participants entering the study in these time spans. The proportion of the 299 participants entering the study during each quartile was 38.8% for the first, 26.4% for the second, 22.1% for the third, and 12.7% for the fourth. Significance was determined with a one-way ANOVA followed by Tukey’s test. In addition, scatter plots with LOESS fit (local regression; span = 0.2), were used to visualize shorter scale variations in stigma.

## Results

### Participants

The characteristics of the study participants (N = 299) are detailed in Table 2. The median time from ETU discharge to study entry was 393 days (IQR: 336–492). The median age of participants was 31 years (IQR: 25–40) and almost half were women (43%). The median number of study visits was 7 (IQR: 6–7); the median days between visits was 97 days (IQR: 83–118) and the median days of on-study follow-up was 597 days (IQR 511–632). Seven (2.3%) participants missed a single study visit during the follow-up period.

**Table 2:** Characteristics of study participants.

		n	percent	median	range
All participants		299	100		
Gender	Male	169	57		
	Female	130	43		
Residence	Monrovia Area	278	93		
	Other	21	7		
Relationship	Single	54	18		
	In a Relationship	240	80		
	Other	5	2		
Education	none	35	12		
	Some primary	55	18		
	Competed primary	22	7		
	Some secondary	70	23		
	Completed secondary	66	22		
	Some tertiary	51	17		
Employment	None	78	26		
	Small business	100	33		
	Casual labor	12	4		
	Farming/fishing	2	1		
	Health care	12	4		
	student	53	18		
	other	43	14		
Age	14–18	12	4	31	14–68
	19–25	72	24		
	26–39	137	46		
	40–59	76	25		
	60+	2	1		
Days from ETU discharge to enrollment		299		393	208–610
Days in study follow-up		299		597	224–725
Number of study visits		299		7	3–9
Days between study visits		1650		97	9–462

### Stigma measured at baseline

**Fig 1:**
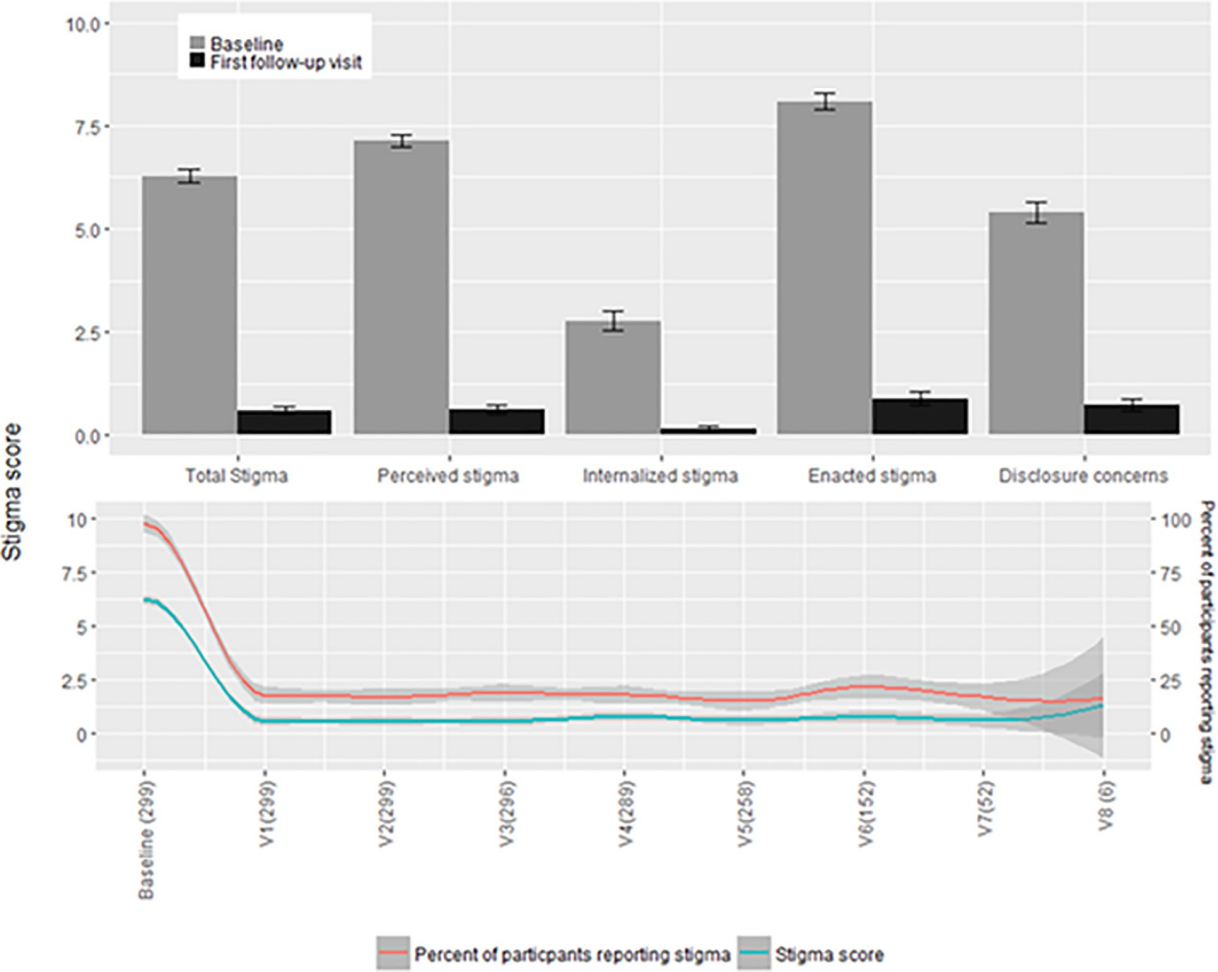
Total and subscale stigma scores at baseline and first follow-up visits. Fig 1: Top-panel: Comparison of mean baseline stigma scores and stigma scores at first follow-up visit for all respondents. Error bars represent standard error. All differences are significant: total stigma [t(505) = 31.88, P<0.0001], perceived stigma [t(599) = 35.61, P<0.0001], internalized stigma [t(330) = 10.70, P<0.0001), enacted stigma [t(563) = 29.65, P<0.0001], disclosure fears [t(472) = 15.69, P<0.0001]. Bottom panel: Scatter plots of the proportion with at least one stigma indicating response across visits and the mean stigma scores for all respondents with local regression lines using LOESS method and span = 0.2. X-axis labels are the study visit followed by the number of participants sampled at that visit. Later study enrollees have not yet reached visits 6, 7 and 8. Gray bands represent 95% confidence intervals. All follow-up visits are significantly different from the baseline-visit (F_8,1941_ = 282.1, p<0.0001), and do not significantly differ from each other (F_7, 1643_ = 1.145, p = 0.33).

At baseline, 98% of participants provided at least one stigma-endorsing response and of those that did the mean stigma score was 6.41 ± 0.14 (IQR: 5–8.75). The mean participant baseline stigma score, reflecting the period from ETU discharge to study entry was 6.28 ±0.15(IQR: 4.38–8.75) (on the 10-point scale) (Fig 1). At baseline, mean stigma sub-scores were highest for enacted stigma (8.08 ±0.19, IQR: 6.67–10.00) followed by perceived external stigma (7.13 ±0.15, IQR: 6.25–8.75), and internalized stigma (2.77 ±0.24, IQR: 0–6.67, F_2, 893_ = 210.2, P<0.0001). The mean disclosure concern sub-scale score at baseline was 5.40 ±0.26 (IQR 0–10).

### Changes in stigma from baseline to follow-up

At the first post-baseline study visit (median of 107 days from baseline, IQR: 97–136), where the survey timeframe was restricted to experiences since the previous study visit, the mean stigma score was 0.60± 0.10 (IQR: 0–0) (t(505) = 31.88, p<0.0001) compared to mean baseline stigma score) (Fig 1). At this time point, 18% of participants provided at least one stigma-endorsing response and their mean stigma score was 3.36 ± 0.34(IQR: 1.25–5.33).

At subsequent follow-up study visits, the mean stigma scores varied little, ranging from 0.28 ± 0.13 to 1.35 ± 1.35 (F_7, 1643_ = 1.145, P = 0.33), as did the proportion endorsing stigma on one or more items.

**Fig 2:**
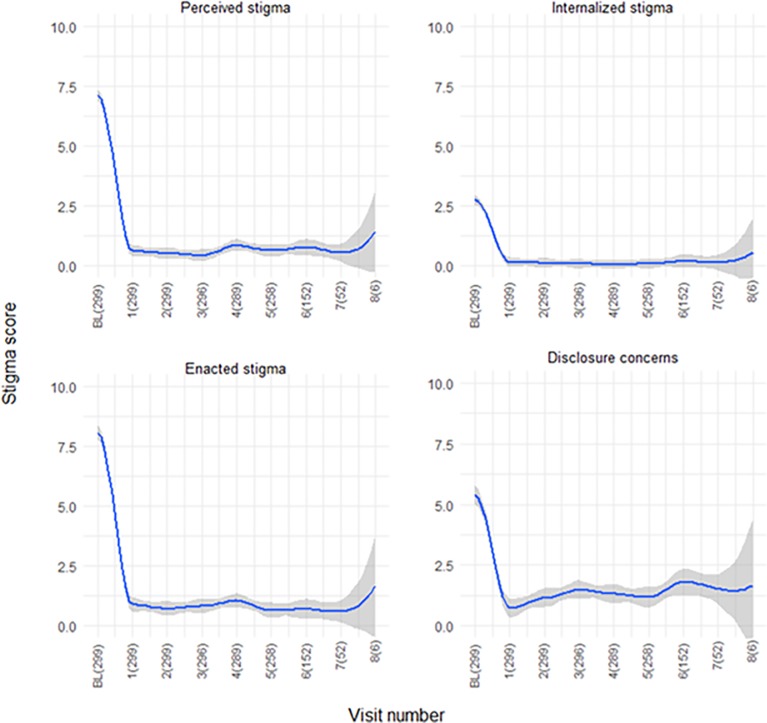
Stigma subscale scores at all study visits. Fig 2: Scatter plots of stigma sub-scores across visits with local regression lines using LOESS method and span = 0.2. X-axis labels are the study visit, followed by the number of participants sampled at that visit. Later study enrollees have not yet reached visits 6, 7 and 8. Gray bands represent 95% confidence intervals. Follow-up visits are not statistically different in the sub-scores: perceived-external stigma (F_7,1643_ = 1.516, p = 0.152), internalized stigma (F_7, 1643_ = 0.924, p = 0.487), and enacted stigma (F_7, 1643_ = 1.14, p = 0.335). The disclosure concern score at visit 6, 1.81, was greater than the score at visit 1, 0.74 (F_7,1643_ = 2.042, p = 0.0469).

Mean aggregated stigma sub-scores at the first post-baseline follow-up visit were highest for enacted stigma (0.89 ± 0.15 [IQR: 0–0]), followed by perceived external stigma (0.61 ± 0.11 [IQR: 0–0]), and were lowest for internal stigma (0.16 ± 0.06 [IQR: 0–0]). Mean sub-scores at subsequent follow-up visits were similar (Fig 2). The disclosure concern mean score at the first post-baseline visit (0.74 ± 0.15 [IQR: 0–0]) was similar to that at the remaining follow-up study visits (which ranged from 0.74 ± 0.15 to 1.81 ± 0.31); nearly half of the participants, over the course of follow-up, reported reluctance to disclose Ebola survivor status.

### Temporal trends in stigma reported at baseline

**Fig 3:**
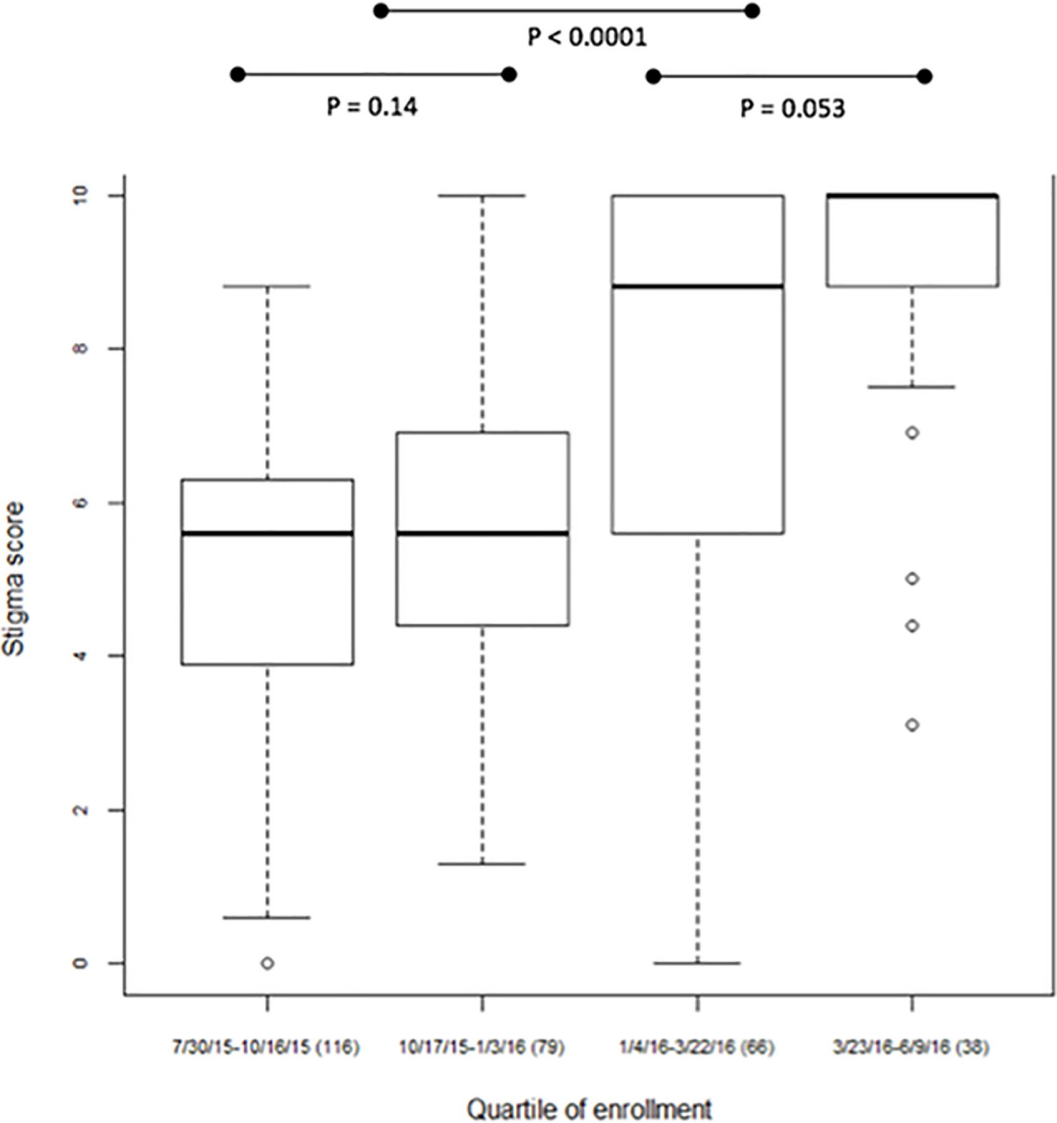
Trend in baseline total stigma scores over time. Fig 3: Comparison in baseline stigma scores of participants enrolled in the different quartiles of enrollment. X-axis labels are the date range for each quartile of enrollment followed by the number of participants recruited in the quartile in parentheses.

**Fig 4:**
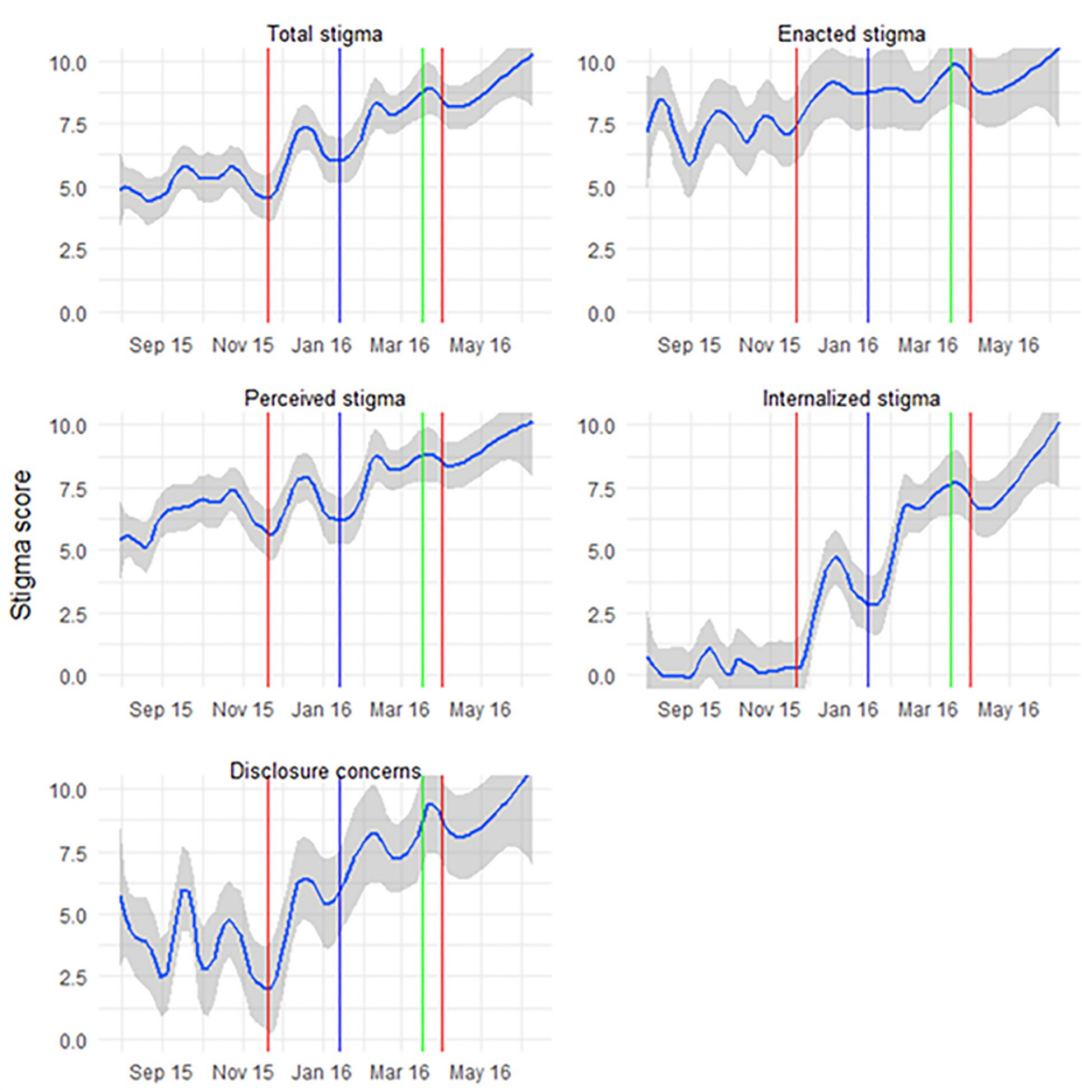
Changes in baseline total and subscale stigma scores during Ebola re-emergence events. Fig 4: Scatter plots of baseline stigma score data with local regression lines using LOESS method and span = 0.2. Vertical lines represent Ebola re-emergence. Gray bands represent 95% confidence intervals.

Mean baseline stigma scores rose over the course of the 12-month period that participants were enrolled (Fig 3). The mean baseline stigma score was 5.05 ± 0.18 (IQR: 3.81–6.25) among the participants entering the study during the first quartile of the enrollment period, and increased to 5.74 ± 0.24 (IQR: 4.38–6.88) in the second, 7.64 ± 0.36 (IQR: 5.53–10.00) in the third, and 8.80 ± 0.32 (IQR: 8.44–10.00) in the last quartile (F_3, 295_ = 38.04, P<0.0001, Fig 1). Increases in participant baseline stigma scores coincided with the November 20^th^, 2015 and April 1^st^, 2016 Liberian Ebola re-emergences, as well as the January 14^th^, 2016 Sierra Leonean re-emergence (Fig 4). However, no such increase was noted after the March 17^th^, 2016 re-emergence in Guinea. Internalized stigma, which had been low in enrollees from July 30^th^, 2015 until the middle of November 2015, increased after the November 20^th^ disease re-emergence (Fig 4, bottom right panel).

## Discussion

In this longitudinal study of a large cohort of Liberian Ebola survivors, most living in or around Monrovia, we found that enacted and external stigmatization was a nearly ubiquitous experience reported by those entering the cohort and persisted for a substantial proportion during study follow-up. These findings are congruent with those reported from smaller and shorter-term studies of Sierra Leonean and Guinean survivors in the months after ETU discharge.[13,14] The main forms of stigma faced by study participants were enacted and perceived external stigma—corroborating the many reports of discrimination, prejudice, and social isolation that arose during the outbreak.[7–10]

The pervasiveness and intensity of stigma reported by the study participants can be expected to have both emotional and physical consequences. Several studies have examined the mental health impact of Ebola survivorship and have found high levels of distress, including anxiety and depression; however, the contribution of stigma to these conditions remains largely unexplored. In persons living with HIV/AIDS, the anxiety and distress imposed by enacted and perceived external stigma have been linked to poor health outcomes^11^ and it has been suggested that the post-Ebola syndrome might be contributed to by somatic manifestations of anxiety and psychiatric disease.[9,10,13] Therefore, while there is no direct evidence that stigma-reducing interventions will improve the mental health of EVD survivors, the known effects of stigma on emotional health and well-being strongly suggest that relief from such stigmatization will be beneficial.

Corrigan and Watson put forth three possible reactions to stigma by those stigmatized; internalization, empowerment to resist the stigma, or indifference towards the stigma.[20] We observed relatively little internalized stigma, suggesting survivor self-worth and confidence were minimally affected by prior Ebola infection. Focus groups recently conducted in Monrovia similarly revealed many survivors to be proud of surviving the disease and empowered to educate their friends and community.^9^ In surveys conducted in Guinea during the height of the epidemic, however, a large majority of respondents reported feeling less confident due to their status as Ebola survivors.[13]

During enrollment, all three heavily impacted countries were declared Ebola-free only to lose that status due to re-emergent disease. Sexual transmission of Ebola by male survivors even long after the initially suggested 3-month period post-EVD, has been documented.[21–22] Coinciding with the time period of enrollment, however, survivors were implicated in disease re-emergence but the mechanism was often either unknown or not disseminated to the public.[23–28] As late as March 2016, the World Health Organization (WHO) reported that re-emergence clusters of EVD could occur since the virus could linger in the eyes, central nervous system, or genital fluids of some survivors.[23]

The baseline results from our study revealed that increases in stigma coincided with EVD re-emergence, and this timeline suggests that concerns regarding the possibility of disease transmission by survivors following these events could be implicated in the increase in reported stigma. In addition, a growing body of evidence of the considerable long-term complications of Ebola infection including possible recrudescent disease–suggesting subclinical infection—may have contributed to the increase in stigma observed over the second half of enrollment. It is notable that, in general, internal stigma, as well as external, enacted and disclosure concern scores assessed at baseline increased following each re-emergence event. This may reflect survivor concern for persistent infectiousness long after recovery and that new cases may have been a result of sexual transmission of the virus from survivors.

In addition to clusters of EVD re-emergence, the general increase in stigma reported at baseline over time may also be explained by the longer interval between ETU discharge and study entry of those enrolled later in the study–which would have afforded more time and opportunity to encounter stigma. This would provide further evidence that stigmatization continued long after the end of the outbreak. Those entering the cohort study later could also have been more stigmatized survivors who were initially reluctant to join the study, and did so only after more time lapsed. However, in an analysis of follow-up visits, stigma from second half enrollees was not higher than first half enrollees, suggesting second half enrollees were not predisposed to higher levels of stigma over time.

Levels of stigma reported at follow-up visits were far less than the stigma reported at baseline visits. As the baseline survey covered the time period from ETU discharge, it is likely that survivors reflected on events that occurred closer to the period after ETU discharge, whereas at follow-up visits, the time period of interest was the interim since the prior study visit. In addition, it is possible that joining a cohort of survivors strengthened connections to the survivor community, empowered survivors, and served as a conduit to accurate information and social support. In focus groups conducted with Liberian survivors, survivor networks were perceived as being extremely important in disease recovery.[9] In addition, a study of re-integration of Guinean survivors showed that joining the Ebola response, especially donating plasma that could be used to treat those with acute EVD, helped social acceptability.[29] Importantly, though the level of stigma reported by the majority of survivors during the course of follow-up was lower than that reported at baseline, many participants suffered at least some persistent long-term stigmatization and reluctance to disclose their Ebola survivor status.

There are limitations that should be considered when interpreting these results. Foremost, those enrolled in this cohort may not be representative of Ebola survivors in Liberia or other nations in the region. The majority of cohort participants reside in urban Monrovia or its environs. Survivors living in more remote rural areas may have different experiences. In addition, the surveying of participants began months after ETU discharge. The period immediately after community re-entry may be when stigmatization was greatest. As there is no validated measure of Ebola-related stigma, we adapted the Berger HIV Stigma Scale, which has been validated for use in persons with HIV infection. While there are important differences between Ebola and HIV in terms of acquisition, infectiousness, clinical manifestations, and social attitudes, there are also a number of similarities including the social isolation of survivors, unfounded or irrational fears of contagiousness, and community and workplace discrimination.[30] Lastly, as the study is on-going, some participants have not yet contributed to later study visits, limiting the number of observations at these time points.

Collectively, our findings highlight the persistence of external and enacted stigma among EVD survivors and a need to develop approaches to prevent and minimize such stigma during and after future outbreaks, perhaps especially in the immediate aftermath of outbreaks. Drawing on the lessons from the stigmatization of those with HIV infection, Davtyan and colleagues recommend several approaches to addressing similar attitudes toward survivors of EVD.[30] These center on both community education and survivor counseling: the recruitment of popular opinion leaders, such as religious and other trusted community figures, to disseminate accurate information and stigma-reducing messaging at a local level, as well as psychological counseling of EVD survivors to develop coping skills, augmented with survivor peer-support. De-stigmatization efforts can also be applied during and in the immediate aftermath of an outbreak. Minimizing the social isolation and social costs of survivors and their families, economic support for the wider community affected by an outbreak, and the development of re-integration programs that recognize the end of transmission risk have been advocated as pre-emptive approaches to mitigate longer term stigmatization. Most all researchers in this area emphasize the need for the inclusion of survivors in developing interventions for Ebola-related stigma.

In conclusion, the majority of survivors of the Liberian outbreak report Ebola-related stigma, primarily enacted and perceived external stigma. Stigmatization was more prevalent among those who entered the study later and, while declining during follow-up, was persistent and often related to disclosure concerns. Spikes in stigma reported by those entering the study often coincided with small clusters of EVD disease re-emergence. To reduce Ebola-related stigma and its subsequent deleterious effects, future Ebola outbreak responses must include stigma-neutralizing interventions during the outbreak and afterward, particularly during any late re-emergence of EVD cases.

## Supporting information

S1 Dataset (XLSX)
